# Content-Based Image Retrieval and Image Classification System for Early Prediction of Bladder Cancer

**DOI:** 10.3390/diagnostics14232637

**Published:** 2024-11-22

**Authors:** Muhammed Yildirim

**Affiliations:** Department of Computer Engineering, Faculty of Engineering and Natural Sciences, Malatya Turgut Ozal University, 44200 Malatya, Turkey; muhammed.yildirim@ozal.edu.tr

**Keywords:** artificial intelligence, bladder cancer, retrieval, feature extraction, diagnosis of disease

## Abstract

Background/Objectives: Bladder cancer is a type of cancer that begins in the cells lining the inner surface of the bladder. Although it usually begins in the bladder, it can spread to surrounding tissues, lymph nodes, and other organs in later stages. Early detection of bladder cancer is, therefore, of great importance. Methods: Therefore, this study developed two systems based on classification and Content-Based Image Retrieval (CBIR). The primary purpose of CBIR systems is to compare the visual similarities of a user-provided image with the images in the database and return the most similar ones. CBIR systems offer an effective search and retrieval mechanism by directly using the content of the image data. Results: In the proposed CBIR system, five different CNNs, two different textural-based feature extraction methods, and seven different similarity measurement metrics were tested for feature selection and similarity measurement. Successful feature extraction methods and similarity measurement metrics formed the infrastructure of the developed system. Densenet201 was preferred for feature extraction in the developed system. The cosine metric was used in the proposed CBIR system as a similarity measurement metric, the most successful among seven different metrics. Conclusions: As a result, it was seen that the proposed CBIR model showed the highest success using the Densenet201 model for feature extraction and the Cosine similarity measurement method.

## 1. Introduction

The bladder is an essential organ of the excretory system that stores urine in the human body. It is located in the lower abdomen, in the middle of the pelvis. The bladder is a muscular sac that can expand and contract due to its flexible structure. The bladder’s primary function is to store urine from the kidneys and ensure it is excreted appropriately [1,2]. Bladder cancer is the second most common malignancy among urological cancers and is a significant health problem worldwide. Bladder cancer is a type of cancer that begins in the cell layer known as the bladder epithelium, especially in transitional cells. The most common histological type clinically is urothelial carcinoma. Other less common types are squamous cell carcinoma and adenocarcinoma. Bladder cancer is approximately three times more common in men than in women and is more common in older ages [3,4,5].

It is known that environmental and genetic factors play an essential role in the etiology of the disease. In particular, smoking is considered one of the most vital risk factors for the development of bladder cancer. In addition, exposure to industrial chemicals, especially aromatic amines, is a significant risk factor for developing the disease. Bladder cancer usually presents with symptoms such as visible hematuria and burning during urination but tends to remain silent in the early stages [5,6,7,8].

Bladder cancer represents a heterogeneous group of diseases in terms of clinical course and response to treatment. Since the risk of tumor recurrence and progression is high in patients with superficial bladder cancer, long-term follow-up is required. Molecular and genetic studies in recent years have revealed subtypes of bladder cancer and the different biological behaviors of these subtypes, promising for the personalization of treatment strategies. Treatment options include surgery, chemotherapy, immunotherapy, and intravesical therapy, and they vary according to the stage and degree of the disease. In this context, early diagnosis of bladder cancer, appropriate treatment management, and regular follow-up programs are critical for the survival and quality of life of patients [9,10,11].

In this study, artificial intelligence-based systems have been developed to treat bladder cancer at an early stage and more efficiently. Artificial intelligence has been used in many areas in recent years, and health is at the forefront of these areas [12,13]. In this paper, both a classification and a CBIR-based system have been developed. The innovative aspects of the study and its contributions to the literature are listed in items.

- In this study, which was conducted to detect bladder cancer, two systems were developed: classification and CBIR-based. In recent years, classification has become one of the most preferred methods in the literature. However, the most significant disadvantage is that the training of the models takes a long time in large datasets, and they cannot produce successful results in multi-class datasets. Therefore, it is crucial to use CBIR-based systems.
- For feature extraction in the CBIR-based system, LBP and HOG architectures were preferred for textural-based models, and Densenet201, GoogleNet, InceptionV3, GoogleNet-Places365, and NasnetLarge architectures were preferred for CNN architectures.
- Feature maps of the architectures were classified in machine learning classifiers, and the highest success was achieved in the Densenet201 + Subspace KNN duo with 99% accuracy.
- The feature extraction model obtained with the Densenet201 was used in feature extraction in the proposed CBIR model, and the similarity and distance measurement methods in our CBIR system were compared.
- This paper examined the performance of seven different architectures and seven different similarity measurement metrics for the proposed CBIR system.
- An average AP value of 0.95302 was obtained by using the Densenet201 feature extraction and Cosine similarity measurement metric.

Section 2 of this study, which was carried out to predict bladder cancer, examined the method and dataset used in the study. Section 3 gave the results of the developed classification and CBIR-based system. Section 4 presented the Discussion section, and Section 5 presented the Conclusion section.

## 2. Materials and Methods

This section examines the dataset used in the study, the developed CBIR-based system, and the classification-based systems. It also includes the sub-methods used in these proposed systems.

### 2.1. Bladder Dataset

**Figure 1:**
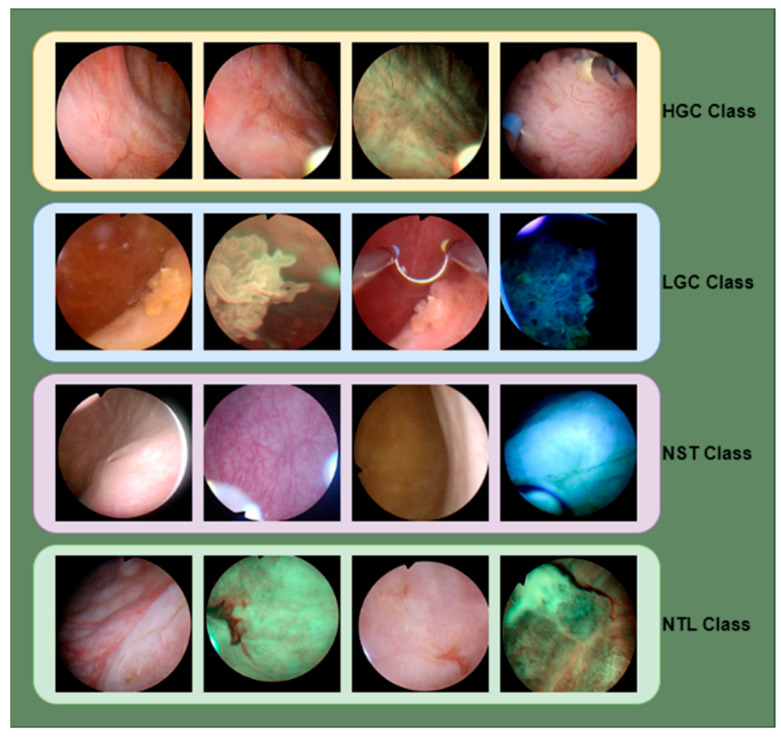
Examples of endoscopic images from the Bladder dataset.

The dataset comprises 1754 bladder endoscopic images from 23 patients who underwent transurethral resection. White light imaging and narrow band imaging techniques were used to create the dataset. The dataset consists of 4 classes. Low-Grade Cancer (LGC) and High-Grade Cancer (HGC) are two classes. In contrast, the other classes consist of cystitis caused by infections or other inflammatory agents, Non-Suspicious Tissue Tumor Lesion (NST), and Non-Tumor (NTL) images [14,15]. There are 469 images in the HGC class, 647 in the LGC class, 504 in the NST class, and 134 in the NTL class. Of the total 1754 images in the classes, 451 images are 300 × 300 pixels, and 1303 images are 350 × 350 pixels. Sample images in the dataset are given in Figure 1.

The original images were studied without preprocessing them into the dataset. The performance achievements obtained on the original dataset were observed.

### 2.2. Structures Used in Proposed Hybrid Systems

In the CBIR system implemented to detect bladder cancer types, feature maps of images in the dataset were first obtained using seven different feature extraction methods. Then, seven different similarity measurement metrics were used for feature extraction. Densenet201 [16], GoogleNet [17], InceptionV3 [18], NasNetLarge [19], GoogleNet-Places365 [20], Local Binary Patterns (LBP) [21], and Histogram of Oriented Gradients (HOG) [22] methods were used for feature extraction.

CNN architectures stand out as a powerful feature extraction method in image processing. CNN architectures learn low-level and high-level features in images through layers. The first layers detect low-level visual features such as edges, corners, and textures, while deeper layers learn object parts and more abstract features. While the convolution process captures local features in the image, pooling layers reduce these features to a more compact form [23,24]. This way, CNNs can be effectively used in tasks such as image classification and content-based image retrieval CBIR. These features extracted in deep layers create more complex and meaningful representations than traditional manually extracted features, which enables deep learning-based models to achieve high performance.

LBP and HOG are two traditional feature extraction methods widely used in image processing and computer vision. LBP represents local textural features by comparing the intensity of each pixel in an image concerning neighboring pixels. The values obtained from pairwise comparisons of pixels are effective in recognizing textural patterns. HOG emphasizes the orientations of edges and corners in an image. It divides the image into cells and extracts histograms of edge orientations in each cell. HOG is a powerful method for capturing edge-based details, especially in object detection and recognition tasks. Both methods provide a better representation by extracting the structural and geometric features of the image [25,26].

In CBIR systems, various similarity measurement methods are used to measure the similarity between the query image and the images in the database. These methods are performed on the feature vectors of the images. Euclid, Manhattan, Cosine, Histogram Intersection, MSE, PSNR, and SSIM are the most commonly used metrics. The CBIR system developed to retrieve bladder cancer images preferred these distance measurement metrics [27].

### 2.3. Developed CBIR System

This study extracted feature maps of images using the Densenet201 architecture from CNN architectures. DenseNet201 is a deep learning model that stands out with its dense connection structure and aims to reduce the weakening gradient problem encountered in the training of deep neural networks. The model consists of 201 layers and is particularly notable for its direct connections between layers; each layer uses the output of all previous layers as input. The architecture of the model consists of four main dense blocks, and at the end of each block, there are transition layers to reduce the number of channels and computational cost. Each dense layer is structured with 1 × 1 and 3 × 3 convolution layers, and the growth rate of the model is maintained by combining feature maps between these layers. The model, which starts with the initial convolution and max pooling layers, is finally terminated with the global average pooling and fully connected layer. Thanks to the parameter efficiency and feature reusability, DenseNet201 optimizes the number of parameters while achieving high accuracy. The proposed model takes input images in 224 × 224 size and produces 1000 features for each image in the fully connected layer [16].

A feature map was extracted from the dataset with the Densenet201 architecture, and a feature vector of 1754 × 1000 size was obtained for 1754 images in the dataset. This feature vector was considered a feature extraction model in our developed CBIR system. The Densenet201 architecture is used for feature extraction of the dataset and the images to be queried in the proposed CBIR system.

**Figure 2:**
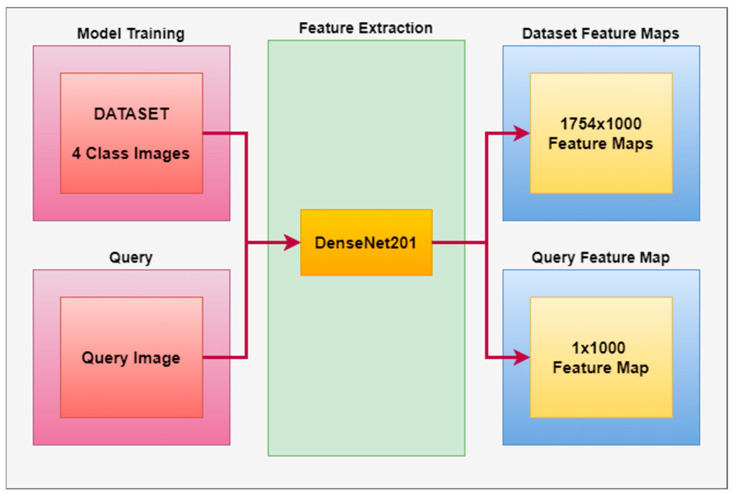
Feature Map Extraction for Proposed CBIR System.

Figure 2 gives the block diagram of the proposed model for feature map extraction of the dataset and the image to be queried in CBIR.

**Figure 3:**
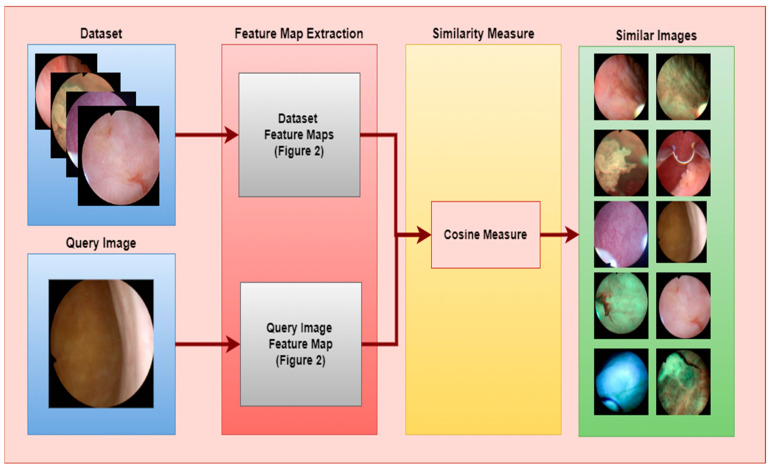
Bladder Cancer Prediction Model for CBIR Systems.

Image search was performed using the feature map obtained in our CBIR system. In the developed model, the feature map of the image to be searched was extracted as in the proposed model, and a comparison was made on the feature map with similarity measurement methods. The comparison with similarity measurement methods was evaluated by measuring the P-R curve with an 11-point sequential access curve. Similarity measurement methods were compared with Euclidean, Manhattan, Cosine, Histogram Intersection, MSE, PSNR, and SSIM methods. In the evaluation made on the 11-point sequential access P-R curve, high performance was achieved with the Cosine similarity method. The block diagram of the proposed CBIR model is given in Figure 3.

**Figure 4:**
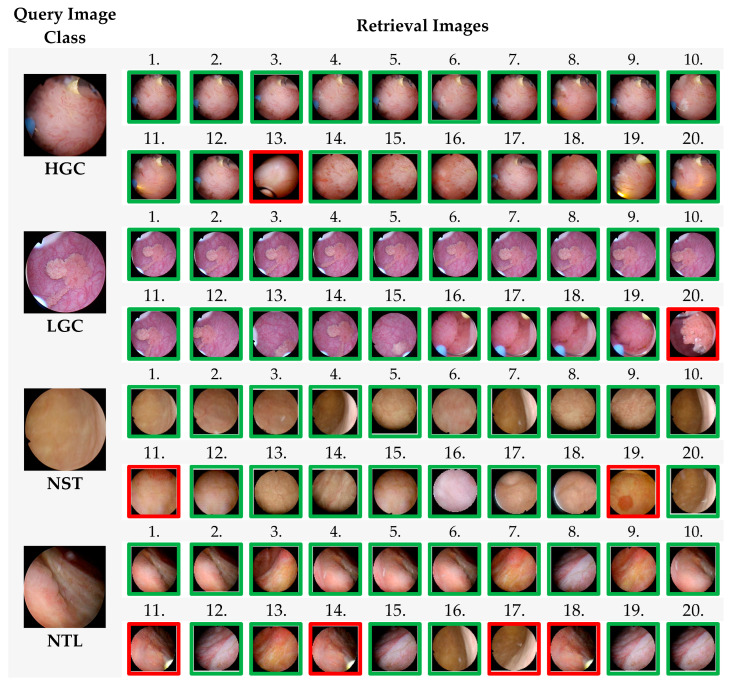
Twenty similar images accessed with the proposed CBIR System.

Figure 4 shows that the proposed CBIR-based model accesses 20 similar images in the dataset. 4 images are randomly selected from the classes in the dataset, and the 20 similar images accessed in the dataset are shown in order. Images accessed in the correct class are indicated with a green frame, and images accessed in the wrong class are indicated with a red frame.

In Figure 4, 20 images similar to the query image belonging to four different classes given for the query from the dataset were accessed sequentially. As a result of the query, the 1st and 12th images and the 14th and 20th images accessed in an image from the HGC class are in the same class, while the 13th image is in a different class. In the LGC class, the first 19 images accessed are in the same class, while the 20th is in a different class. In the NST class, the 11th and 19th images accessed are in a different class, while the others are in the same class. In the NTL class, the images in the 11th, 14th, 17th, and 18th places are different, while the others are in the same class.

## 3. Application Results

This study used the parameters as constant in the deep learning models and classifiers. The same values were used in all architectures and classifiers. The results of these studies were obtained using the MATLAB 2024b program on a computer with an Intel i5 processor (Santa Clara, CA, USA), 16 GB RAM, and a Windows 10 64-bit operating system.

### Deep Model and Classification Results

This study used seven different models for feature extraction in the classification and CBIR system. The same training parameters were used to accurately compare the training results on these deep and textural-based models.

In the study, Densenet201, InceptionV3, GoogleNet, NasNetLarge, GoogleNet-Places365, LBP, and HOG architectures were compared. In CNN architectures, feature maps are taken from the last fully connected layer. The number of features obtained for each image in CNN architecture is 1000. In LBP architecture, the number of features obtained for each image is 2891, while in HOG, it is 1296. The feature sets obtained from this architecture were given to the traditional machine learning classifiers separately, and the classification was conducted. The accuracy values given by the classifiers are shown in Table 1.

**Figure 5:**
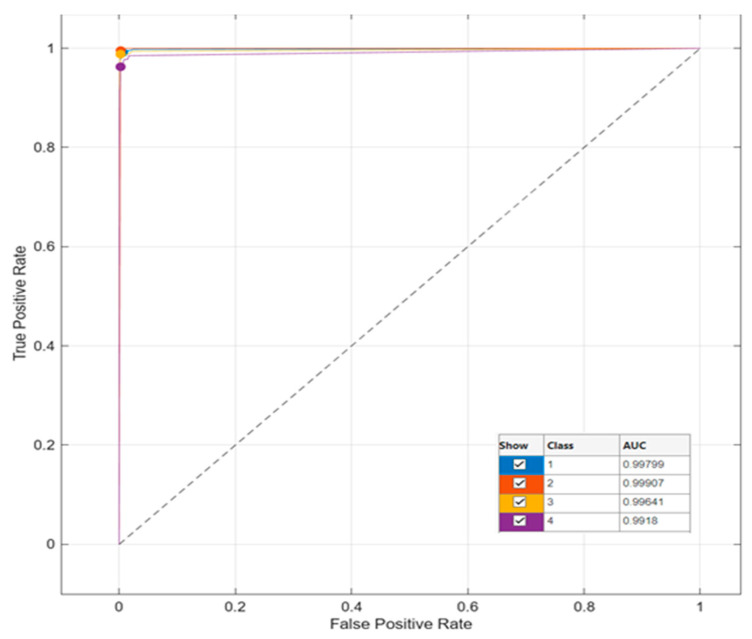
AUC-ROC Curve of DenseNet201 + Subspace KNN duo.

The highest performance with 99.0% accuracy was achieved in the Subspace KNN classifier with the DenseNet201 architecture obtained in Table 1. The accuracy rate was 95.8% in the InceptionV3 architecture, 97.0% in the GoogleNet architecture, 96.2% in the NasNetLarge architecture, 96.5% in the GoogleNet-Places365 architecture, 97.9% in the LBP architecture, and 94.5% in the HOG architecture. In this study, conducted for bladder cancer detection, the highest accuracy value of 99.0% was obtained in the DenseNet201 + Suspace KNN duo. The AUC-ROC curve of the DenseNet201 architecture is given in Figure 5.

The order of the images accessed in CBIR systems is important. In such CBIR systems, evaluations are made with standard 11-point P-R graphs. In the CBIR system we developed, a P-R graph is given for 20 images of a randomly searched image in each class. The performance of the similarity measurement methods, Euclid, Manhattan, Cosine, Histogram Intersection, MSE, PSNR, and SSIM methods, was evaluated in the P-R graph.

**Figure 6:**
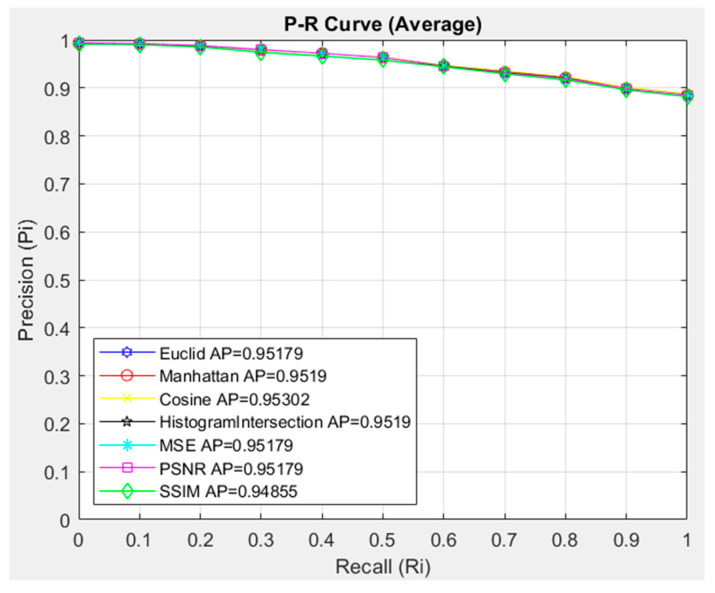
P-R Plot of Similarity Measurement Methods of CBIR Model with DenseNet201.

In this study, the interpolated 11-point sequential access was evaluated with the P-R curve and by calculating the Average Precession (AP) value. The images in each class were queried one by one and the average P-R curve was obtained. In the CBIR system, the images in each class were queried separately and 20 images were accessed on the system. The images queried in each class, the images accessed were evaluated, and the average P-R curve was created. After the P-R curve of the four classes was evaluated separately, 1754 images in the dataset were queried and the Average P-R curve of the 20 images accessed in each query was calculated and evaluated. In Figure 6, using the DenseNet201 feature extraction architecture with the highest accuracy, each of the 1754 images in 4 classes was evaluated with an 11-point sequential access P-R graph for accessing 20 images in the CBIR system. Since 20 images were accessed for each of the 1754 images in the dataset, a total of 35,080 image accesses were compared. In this comparison, the performance of the Euclidean, Manhattan, Cosine, Histogram Intersection, MSE, PSNR, and SSIM similarity and distance measurement methods were compared, and the most successful similarity measurement method to be used in the proposed CBIR system architectures was evaluated.

**Figure 7:**
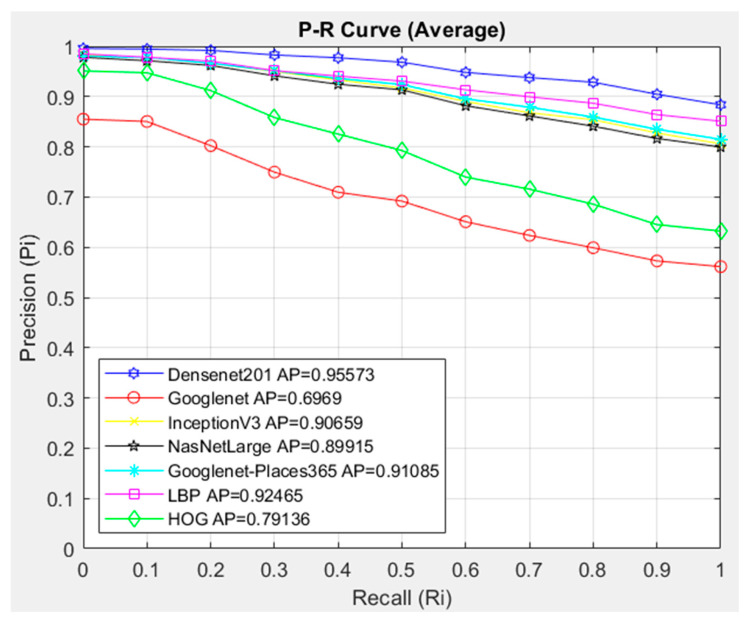
Average P-R curve of HGC class.

Figure 6 compares the similarity measurement methods that should be used in the proposed CBIR system with the DenseNet201 architecture. When the general performance is examined, it is seen that the similarity and distance measurement methods are similar to each other. It was evaluated that the most successful similarity method is Cosine, with an AP value of 0.95302. The SSIM similarity measurement method showed the lowest success with an AP value of 0.94855. In this case, when the general average of the similarity and distance measurement metrics for our proposed CBIR system with the DenseNet201 architecture, which shows the highest accuracy in feature extraction, is evaluated, it is seen that the metrics are obtained with very close AP values, and the Cosine similarity measurement metric showed the highest success with an AP value of 0.95302 among these metrics. It was evaluated that the Cosine model is the most suitable for use in the dataset for the CBIR system. The Cosine similarity measurement metric will be used to compare the success of the architectures used for feature extraction in the CBIR system. In Figure 7, the interpolated 11-point sequential access P-R curve of the images in the HGC class is compared with the architectures in the proposed CBIR system using Cosine. 469 images in the HGC class were queried in the CBIR system, and access to 20 similar images in the dataset was provided. Since 20 similar images were accessed for each image queried in the HGC class, 9380 were accessed.

Figure 7 compares the architectures with the Cosine similarity measurement method. In the CBIR system, the DenseNet201 architecture showed the highest success with AP = 0.95573 in the images in the HGC class. The GoogleNet architecture had the lowest HGC class average success with AP = 0.6969. When the P-R graph is examined, it is seen that the DenseNet201 architecture is more successful in image access than the other architectures in all cases in the Recall (0–1) range. The GoogleNet architecture, on the other hand, shows much lower success than the other architectures. NasnetLarge, GoogleNet-Places365, and Inceptionv3 architectures provided access with a value close to each other in all cases.

Compared with the 11-point sequential access P-R curve interpolated in the HGC class, the DenseNet201 architecture was more successful with the Cosine metric with the AP = 0.95573 value.

**Figure 8:**
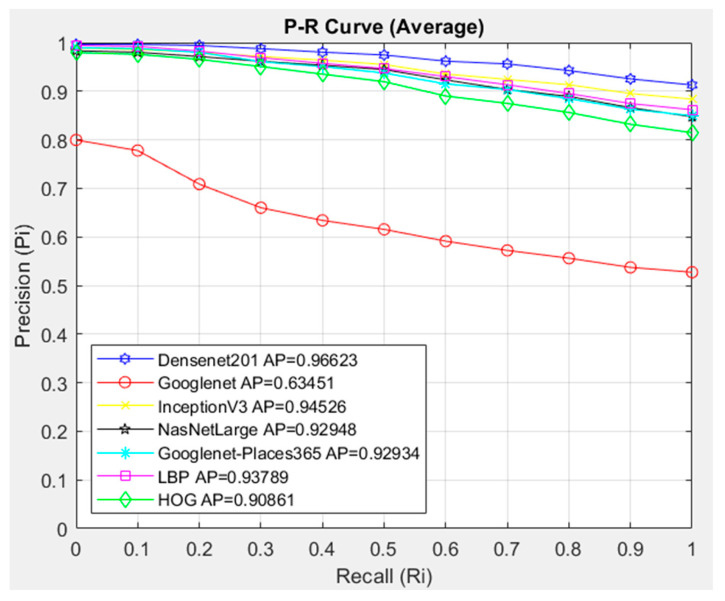
Average P-R curve of LGC class.

In Figure 8, the interpolated 11-point sequential access P-R curve of the images in the LGC class is compared with the architectures in the proposed CBIR system using Cosine. The CBIR system queried 647 images in the LGC class, and access to 20 similar images in the dataset was provided. Since 20 similar images were accessed for each image queried in the LGC class, 12,940 images were accessed.

In Figure 8, the architectures are compared with the Cosine similarity measurement method. In the CBIR system, the DenseNet201 architecture showed the highest success with AP = 0.96623 in the images in the LGC class. The lowest success in the LGC class average was shown by the GoogleNet architecture with AP = 0.63.451. When the P-R graph is examined, the Densenet201 architecture showed a more successful slope than the other architectures in all cases in the range of R = (0..1). The GoogleNet architecture was clearly less successful than the other architectures. Although other architectures other than GoogleNet showed success close to the Densenet201 architecture up to approximately R = 0.2, a decrease was observed after the R = 0.2 case.

In the comparison with the interpolated 11-point sequential access P-R curve in the LGC class, the DenseNet201 architecture was more successful with the Cosine metric with the AP = 0.96623 value.

**Figure 9:**
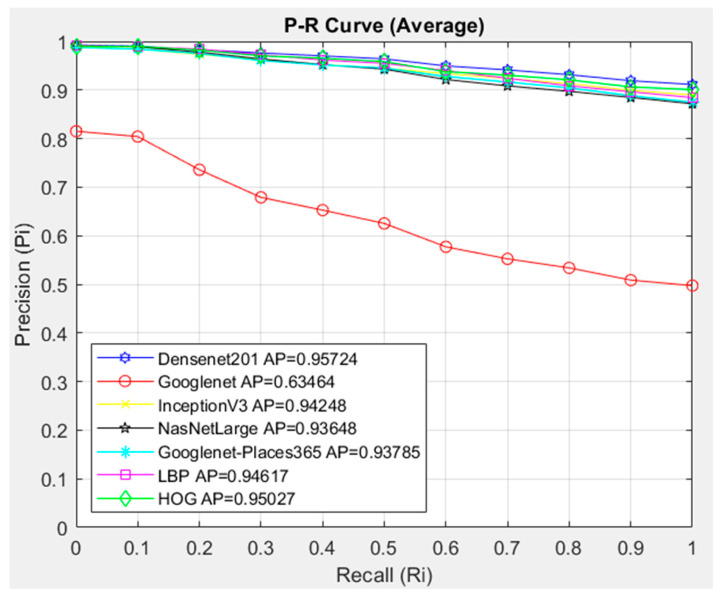
Average P-R curve of NST class.

In Figure 9, the interpolated 11-point sequential access P-R curve of the images in the NST class is compared with the architectures in the proposed CBIR system using Cosine. 504 images in the NST class were queried in the CBIR system and access was provided to 20 similar images in the dataset. Since 20 similar images were accessed for each image queried in the NST class, a total of 10,080 images were accessed.

Figure 9 compares the architectures with the Cosine similarity measurement method. In the CBIR system, the DenseNet201 architecture showed the highest success with AP = 0.95724 in the images in the NST class. The GoogleNet architecture showed the lowest success in the NST class average with AP = 0.63464. When the P-R graph is examined, it is seen that the other architectures except GoogleNet provide similar image access in all cases in the R = (0.1) range. In these architectures that follow each other closely, it is seen that the Densenet201 architecture provides slightly more accurate image access in all cases.

Compared with the 11-point sequential access P-R curve interpolated in the NST class, the Densenet201 architecture was more successful with the Cosine metric with the AP = 0.95724 value.

**Figure 10:**
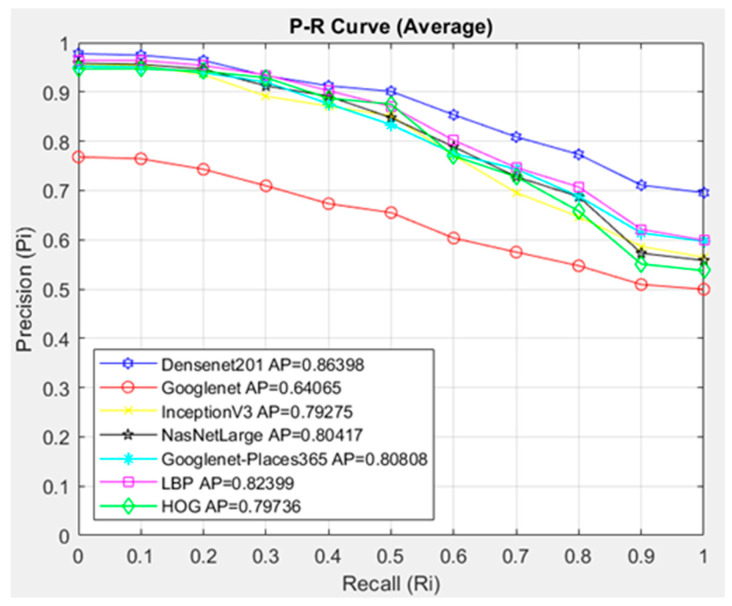
Average P-R curve of NTL class.

In Figure 10, the interpolated 11-point sequential access P-R curve of the images in the NTL class is compared with the architectures in the proposed CBIR system using Cosine. The CBIR system queried 134 images in the NTL class, and 20 similar images in the dataset were accessed. Since 20 similar images were accessed for each image queried in the NTL class, 2680 were accessed.

Figure 10 compares the architectures with the Cosine similarity measurement method. In the CBIR system, the DenseNet201 architecture showed the highest success with AP = 0.86398 in the images in the NTL class. The GoogleNet architecture showed the lowest success in the NTL class average with AP = 0.64065. When the P-R graph is examined, it is seen that the DenseNet201 architecture provides more successful image access than the other architectures in the R = 0.4 state and later.

When compared with the interpolated 11-point sequential access P-R curve in the NTL class, the DenseNet201 architecture was more successful with the Cosine metric, with the AP = 0.86398 value.

**Figure 11:**
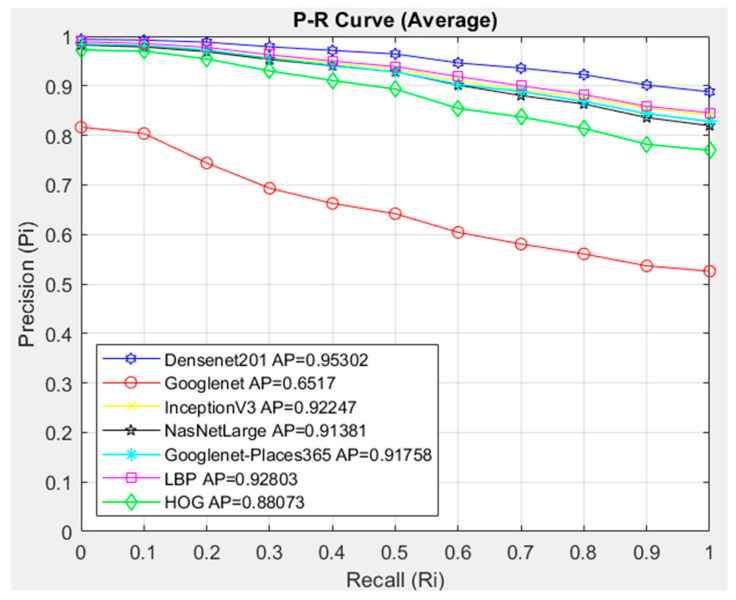
Overall Average P-R curve of the dataset.

When the images in the HGC, LGC, NST, and NTL classes in the dataset were evaluated separately in our CBIR system, the DenseNet201 architecture and the Cosine similarity measurement metric achieved success in all classes in feature extraction in the CBIR system according to the interpolated 11-point sequential access P-R curve, InceptionV3, HOG, LBP, NasNetLarge, GoogleNet and GoogleNet-Places365 architectures showed low success in all classes according to the proposed model. GoogleNet architecture showed the lowest success in all classes. In Figure 11, the interpolated 11-point sequential access P-R curve of all images in the dataset is compared with the architectures using Cosine. A total of 1754 images in the dataset were queried in the CBIR system, and 20 similar images were accessed. Since 20 similar images were accessed for each image queried in the dataset, a total of 35,080 images were accessed.

After comparing the average of all classes, when the general average is examined in Figure 11, as in all classes, the DenseNet201 architecture was more successful in our proposed CBIR system compared to other models. Although InceptionV3, NasNetLArge, GoogleNet-Places365, and LBP architectures showed similar success, it is seen that they showed lower success in image retrieval compared to the Densenet201 architecture. The GoogleNet architecture showed the lowest success. In Figure 11, the use of the Cosine similarity measurement metric with the DenseNet201 feature extraction architecture was more successful in similar image retrieval with AP = 0.95302.

## 4. Discussion

Bladder cancer continues to occur worldwide and has significant impacts on patient quality of life, morbidity, mortality, and cost to the healthcare system. Bladder cancer surveillance consists of periodic white light cystoscopy and urine cytology. However, both diagnostic methods have some limitations. Therefore, new, innovative diagnostic tests are needed to improve the management of recurrent bladder cancer. This requires both additional cost and workload. Therefore, it is important to perform bladder cancer detection with artificial intelligence-based systems [28,29].

In the literature, CBIR systems and classification problems are applied to different topics in different fields [30,31,32]. In this study, which was carried out for the detection of bladder cancer types, both classification processes were performed using CNN and textural-based models and a CBIR-based system was developed. In this developed system, access to similar images is provided by extracting feature maps with CNN and textural-based models. In this study, the performance of our proposed model was compared with CNN architectures Densenet201, InceptionV3, NasNetLArge, GoogleNet-Places365, GoogleNet, and textural-based models LBP and HOG. DenseNet201 was used for feature extraction in our proposed model. Similarity measurement metrics were compared using the DenseNet201 architecture, which gives 99.0% accuracy in machine learning classifiers of the images queried in our CBIR system and included in the database. By obtaining the general average of the P-R curve of the dataset, it was observed that the Cosine similarity measurement method with the Dense201 model gave more successful results than other similarity measurements. In this study, access to the 20 images with the closest similarity is provided. The performance measurement of the images accessed as a result of the queried image was evaluated by calculating AP with the interpolated 11-point sequential access P-R curve.

The interpolated 11-point sequential access P-R curve was evaluated separately for four classes and for all images in the dataset. When the architectures were evaluated with the Cosine metric in the proposed CBIR system in the HGC, LGC, NST, and NTL classes and in the dataset in general, as in machine learning classifiers, the DenseNet201 architecture was more successful than other architectures in image retrieval in the CBIR system. The DenseNet201 architecture with the Cosine method showed great success in the CBIR system.

The highest success was achieved in the CBIR system with the Cosine method in the Densenet201 architectural model; with AP = 0.95573 in the HGC class, AP = 0.96623 in the LGC class, AP = 0.95724 in the NST class and AP = 0.86398 in the NTL class compared to other architectures. In general, when we evaluate all the images in the dataset in the CBIR system, the highest performance was achieved with AP = 0.95302 using the Cosine similarity method and the Densenet201 architecture. The biggest limitation of this study, which was carried out for bladder cancer detection, is the small number of data and the lack of data collected from different centers. In order to strengthen the study, data should be collected from different regions and different centers, and the study should be multi-centered. Another limitation of the study is that the developed system may not be able to effectively detect early-stage bladder cancer because a high-grade tumor may already be muscle-invasive.

## 5. Conclusions

Bladder cancer is a type of cancer that develops in the cells lining the inner surface of the bladder. Treatment options may include surgery, radiotherapy, chemotherapy, and immunotherapy, depending on the tumor stage. It is vital to detect the tumor in its early stage successfully. In this study, which was carried out for automatic early bladder cancer prediction, two different systems were developed. In the first system developed, the classification process was performed. In the classification-based system, an accuracy value of 99% was obtained. The second system implemented for bladder cancer prediction is a CBIR-based system. The most important feature of CBIR-based systems is that they can produce successful results in datasets with many data and classes. In the proposed CBIR-based system, the AP value was 0.95302. This value was brought to 20 images for each query. Successful results were obtained in bladder cancer classification in both developed artificial intelligence-supported systems. Thanks to the developed artificial intelligence-supported system, it will allow automatic detection of bladder cancer types at an early stage.

## Figures and Tables

**Table 1:** Comparison of the architectures.

	SUBSPACE KNN	QUADRATİC SVM	CUBİC SVM	MEDIUM GAUSSIAN SVM	FINE KNN	SUBSPACE DISCRIMINANT
DENSENET201	99.0	97.8	98.1	97.7	98.2	97.2
INCEPTIONV3	95.8	95.3	96.5	94.9	95.9	95.0
GOOGLENET	97.0	96.0	96.1	95.6	97.0	95.7
NASNETLARGE	95.4	96.1	96.2	95.2	95.7	96.0
GOOGLENET-PLACES365	96.2	95.7	96.3	94.4	96.5	93.9
LBP	97.7	95.3	96.3	95.3	97.9	82.6
HOG	94.5	92.7	94.2	90.9	94.0	89.8

## Data Availability

A public dataset was used in the study.
